# Vitamin D Interactions with Soy Isoflavones on Bone after Menopause: A Review

**DOI:** 10.3390/nu4111610

**Published:** 2012-11-06

**Authors:** Clara Y. Park, Connie M. Weaver

**Affiliations:** Department of Nutrition Science, Purdue University, 700 W State St, West Lafayette, IN 47907, USA; Email: cypark@purdue.edu

**Keywords:** vitamin D, phytoestrogen, soy isoflavones, bone, postmenopause

## Abstract

Vitamin D is known to increase Ca absorption in adults. However, the threshold vitamin D status to benefit Ca absorption is lower than the target vitamin D status for higher bone mineral density and lower fracture risk, pointing to another pathway for vitamin D to benefit bone. One possibility is by affecting osteoblast and osteoclasts directly. Vitamin D-related bone metabolism may also be affected by soy isoflavones, which selectively bind to the estrogen receptor β and may reduce bone loss in postmenopausal women. We discuss a possible synergistic effect of soy isoflavones and vitamin D on bone by affecting osteoblast and osteoclast formation and activity in postmenopausal women.

## 1. Introduction

Osteoporosis, “the thinning of the bone tissue and loss of bone density over time” [1], is a major cause of fractures that accompany morbidity and complications that lead to death, especially in older people. Twenty-four percent of women over 50 years who experience hip fractures die within one year [2]. Even for those who survive, quality of life drastically decreases as most become home-bound or require support for daily activities. In the United States, approximately 10 million people were affected by osteoporosis in 2006, and the rate of hospitalization involving injury likely due to osteoporosis increased 55% in 2006 since 1995 [3]. Osteoporosis-related fracture costs in 2005 were estimated at $19 billion and this figure is estimated to increase to $25.3 billion by 2025 [2]. 

Older adults, especially postmenopausal women, are more susceptible to osteoporosis due to estrogen deficiency which promotes bone resorption and inadequate intake of calcium (Ca) and vitamin D, two important nutrients to protect bone health [4]. The traditionally known mechanism for vitamin D to benefit Ca metabolism is through increasing Ca absorption. However, recent data and meta-analyses reveal that the vitamin D level that benefits Ca absorption is inconsistent with the level that optimizes bone mineral density (BMD), which is directly correlated to osteoporosis and hip fracture risk [5]. Similar to Ca and vitamin D, other nutrients may also have an interactive effect on bone health, especially during estrogen deficiency. One proposed mechanism of vitamin D benefiting BMD may be through a synergistic effect with phytoestrogens. In this review, we will briefly discuss the knowledge gaps and possible mechanisms of vitamin D and phytoestrogens on bone health. Among the wide variety of phytoestrogens, we will focus on soy isoflavones genistein and daidzein, as relatively more research has been reported on these compounds.

## 2. Vitamin D and Ca Absorption

The effect of vitamin D on Ca absorption has been well understood and thought to be the major pathway to affect bone health. Vitamin D can be obtained through oral intake or cutaneous synthesis when UV reacts with 7-dehydrocholesterol. Vitamin D is converted to 25-hydroxyvitamin D (25(OH)D) in the liver. 25(OH)D is known to be the status marker of vitamin D, due to its longer (3–4 weeks) half-life. Parathyroid hormone (PTH) and 1,25-dihydroxyvitamin D (1,25(OH)_2_D), the active form of vitamin D, signal the regulation of Ca homeostasis. Ca metabolism is traditionally known to be regulated mainly through absorption, reabsorption, and resorption in the intestine, kidney, and bone, respectively, primarily to control serum Ca concentration. When Ca intake is low, the drop in serum Ca concentration is detected by the calcium sensing receptors on the parathyroid gland. This triggers parathyroid hormone secretion, which induces hydroxylation of 25(OH)D to 1,25(OH)_2_D, bone resorption, and renal reabsorption of Ca. Elevated serum 1,25(OH)_2_D levels stimulate the transcription of genes regulating Ca absorption in the intestine, such as TRPV6, calbindinD_9k_, and PMCA1b [6], and reabsorption in the kidney, *i.e.*, TRPV5 and TRPV6, calbindinD_28k_ and PMCA. The effect of 1,25(OH)_2_D on intestinal gene transcription is more effective than renal gene transcription, and consequently Ca absorption is more affected by circulating 1,25(OH)_2_D than reabsorption. As vitamin D and 25(OH)D are precursors of 1,25(OH)_2_D, it is thought that higher vitamin D intake and higher serum 25(OH)D status, indicating adequate substrate for 1,25(OH)_2_D production, will lead to increased Ca absorption. As a result, when dosed orally or subcutaneously with 1,25(OH)_2_D, Ca absorption increased in rats and humans [7,8]. Increased 1,25(OH)_2_D and serum Ca concentration provide a negative feedback on PTH release so that serum Ca concentration is maintained within a tight window. Once serum Ca is maintained, excess Ca from absorption is deposited in the bones or excreted through urine as PTH is suppressed. Through this process, vitamin D can stimulate Ca absorption, the primary step to increase BMD.

Clinical trials have pointed to a threshold 25(OH)D level of ~20 nmol/L to maximize vitamin D-mediated Ca absorption in adults. Previous research using crude methods resulted in a relatively large increase (65% higher than baseline) in fractional Ca absorption with increased vitamin D status (36.5 nmol/L increase) [9]. However, cross-sectional data using the double isotope gold-standard method resulted in Ca absorption to be significantly low only when 25(OH)D was below 20 nmol/L in adults [10,11,12]. Intervention trials also using the double isotope method found no difference in Ca absorption with vitamin D supplementation [13] or a minimal (absolute 3%) increase despite the 200% increase in 25(OH)D [12] in women with mean baseline vitamin D status over 50 nmol/L. In addition, serum concentration of the active vitamin D metabolite, 1,25(OH)_2_D, is positively correlated with Ca absorption [14,15], but in many cases vitamin D supplementation does not increase circulating 1,25(OH)_2_D concentration [7]. Though vitamin D is essential for Ca absorption at levels below 20 nmol/L, it is possible that vitamin D can benefit bone at higher levels through other mechanisms.

## 3. Vitamin D and BMD

Despite the 20 nmol/L threshold for maximal vitamin D-activated Ca absorption, much data has been presented that higher vitamin D status is required to maximize BMD in adults. Vitamin D status and BMD were positively correlated in monkeys that received oophorectomy [16] and postmenopausal women. Bischoff-Ferrari and colleagues [17] report that serum 25(OH)D status was positively related to BMD. The recent Recommended Dietary Allowances (RDA) for vitamin D for those over 50 years (51–70 years: 600 IU/day, 71 years and older: 800 IU/day) were established based on vitamin D supplementation doses that decreased fracture risk [18]. The target serum 25-hydroxyvitamin D (25(OH)D) level was 50 nmol/L to meet 97.5% of the populations needs. When supplemented with Ca and vitamin D, but not Ca only, BMD was protected in adults with lower (<68 nmol/L) baseline 25(OH)D levels compared to placebo [13]. Even Finish adolescent girls with low baseline vitamin D status had a larger increase in femoral BMC with vitamin D supplementation [19], despite the negative relationship of vitamin D status and Ca absorption in adolescents reported by others [20,21,22]. Therefore, vitamin D may benefit bone independent of Ca absorption.

Increasing evidence has emerged on the local action of vitamin D. The VDR and 1α-hydroxylase (1αOHase) activity has been discovered in bone cells [23]. It has been known that 1,25(OH)_2_D bound to the VDR induces osteoblast, the bone-forming cell, function by activating collagen type 1α, osteocalcin and alkaline phosphatase gene transcription [24]. 1,25(OH)_2_D also inhibits Runx, which stimulates differentiation of mesenchymal progenitor cells to pre-osteoblasts, and pre-osteoblasts to osteoblasts. However, mature osteoblasts are activated by 1,25(OH)_2_D, and the receptor for activation of NFkB ligand (RANKL) binds to RANK on pre-osteoclasts. As a result, osteoclastogenesis occurs and bone resorption is increased. In mice, overexpression of VDR in mature osteoblasts increased bone formation and decreased bone resorption in tibial cortical bone and vertebral trabecular bone, respectively [25]. Also, a decrease in osteoclast and osteoid surface and width, mineralizing surface, and increase in formation period was observed as serum 25(OH)D concentration increased, regardless of circulating 1,25(OH)_2_D levels [26]. Much research is still needed to understand the direct effects of vitamin D on bone metabolism.

The effect of vitamin D on BMD also has a threshold and the effect may vary with Ca intake. At serum 25(OH)D levels beyond 104 nmol/L, Fleet *et al.* [27] reported no effect of vitamin D on BMD in young rats regardless of Ca intake (0.25% or 0.5%). At moderate intakes of Ca (0.5%) and realistic doses of vitamin D (range 25 to 1000 IU/kg diet), vitamin D increased trabecular BMD, BMC, and bone volume, but not femur BMD in growing male rats [28]. In young female rats with low Ca intakes (0.1%), vitamin D intake reduced trabecular number, thickness, and percent cancellous bone [29]. However, rats fed a normal (0.5%) Ca diet were not affected by vitamin D on any of these parameters. In older men and women, only those with Ca intakes lower than the median (716 mg/day) had a relationship between serum 25(OH)D and hip BMD, serum Ca, and serum PTH at 6 and 12 months [30]. Some studies show an increase of BMD and decreased fracture risk when supplemented with both Ca and vitamin D compared to placebo [13,31,32] but this combined treatment was not significantly different from the effect of a single nutrient effect [13,33]. Though the individual benefits of Ca and vitamin D are not always conclusive, the benefit of the combination of Ca and vitamin D supplementation on bone is evident [34].

## 4. Soy Isoflavones and Bone

Some plant-derived compounds that have structural similarity to estrogen, phytoestrogens, are able to bind to the estrogen receptor (ER). The results of phytoestrogen interventions on bone health outcomes vary due to the type of phytoestrogen. Therefore, in this paper we will focus on soy-derived isoflavones genistein and daidzein. The decrease of estrogen [35,36,37] or the impaired responsiveness of bone to low sex steroid concentrations [38] during aging, and especially menopause, negatively affects bone health. Hormone replacement therapy (HRT) prevents bone loss and increases bone mass in postmenopausal women [39], but with additional side effects such as invasive breast cancer [40]. This peaked interest on the effectiveness of phytoestrogens on bone health promotion. Phytoestrogens bind to both α- and β-form ER but preferentially bind to the β-form ER (ERβ). The ERβ is thought to have anti-proliferative effects, thus being a possible target pathway to prevent reproductive cancer while enhancing bone health in post-menopausal women. Most genistein treatment effects disappeared from rat mandibular condyle osteoblasts when ERβ was silenced [41]. Thus, phytoestrogens may protect bone by binding to the ER, especially in estrogen-deficient post-menopausal women.

The effects of isoflavones on bone are thought to be through affecting the bone cells directly, rather than affecting Ca absorption. This is based, in part, on the lack of dose responsive effect of dietary genistein or daidzein in trans-epithelial transport of Ca in ERβ-expressing Caco-2 cell cultures [42]. Caco-2 cells responded to genistein and daidzein similarly to 17β-estrogen with no effect on calbindinD_9k_, an intestinal Ca transport protein [43]. Furthermore, increasing levels of soy isoflavones had no effect on Ca absorption in postmenopausal women [44,45]. On the other hand, it has been shown that isoflavones reach the bone tissue [46]. Low-dose daidzein induced nucleus ERβ, while 17β-estrogen increased ERα in young piglet bone cells [47]. Genistein also increased ERβ expression in rat mandibular subchondrial bone [41]. By binding to ERβ in osteoblastic cells *in vitro*, phytoestrogens induce production of osteoprotegerin (OPG). OPG competes with RANKL and prevents maturation of pre-osteoclasts and thus, resorption [48]. In piglets, daidzein increased osteoblast differentiation, secretion of OPG and RANKL, and bone mineralization more potently than the same dose of 17β-estrogen [47]. In porcine bone marrow, daidzein suppressed 1,25(OH)_2_D-induced TRAP-positive multi-nucleated cell formation, decreased resorption activity, and increased ER expression and apoptosis through caspase-8 and caspase-3 fragmentation in mono-nucleated cells [49]. In humans, genistein supplementation for 1 and 2 years in osteopenic post-menopausal women resulted in a greater decrease in soluble RANKL/OPG by increasing OPG and decreasing soluble RANKL [50]. These evidences imply that genistein and daidzein induce ERβ transcription and binding and reduce mature osteoclastogenesis, and hence bone resorption.

The effect of soy isoflavones on bone in rodent and human trials has mixed results. Genistein positively affected rat mandibular subchondral bone BMD, bone volume, and trabecular bone in a dose-dependent manner [41]. In OVX rodents, genistein supplementation improved femoral total and trabecular BMD [51]. Mathey *et al.* [52] report the benefits of genistein, daidzein, and equol, the metabolite of daidzein, on total and metaphyseal femoral BMD are similar in OVX rats. The effect of genistein and daidzein on bone has also been reported to be similar by others, but an uterotrophic effect of equol has additionally been observed [53]. Others report equol supplementation improved femur calcium, but trabecular structure was diminished and uterotropic effects were also seen at higher doses [54]. In humans, osteopenic postmenopausal women receiving 54 mg of genistein/day for 24 months were protected from bone loss at the lumbar spine and femoral neck [55]. In a shorter 6 month study, 90 mg/day of soy isoflavones protected postmenopausal women against lumbar spine bone loss [56]. Commercial soy isoflavone supplement intake (150–220 mg/day, containing daidzin, glycitin, genistin compounds) for 50 days suppressed bone resorption [43] while up to 135 mg/day of soy isoflavones (daidzin, glycitin, genistin compounds and puerarin, formononectin, or biochanin A) did not show any benefit on bone turnover assessed by urinary excretion of ^41^Ca from pre-labeled bone in a similar population [42]. Others report 2 years of 120 mg/day soy isoflavone supplementation in healthy postmenopausal women protected against whole body bone loss, but not specific sites, such as lumbar spine, femoral neck, or total hip [57]. Neither Taiwanese post-menopausal women [58], nor US women within 1–5 years of menopause [59] benefitted from 2 years of soy isoflavone supplementation. The inconsistent results in human studies compared to rodent research may partially be to due the diverse genetic and environmental factors that contribute to the individual capacity of producing equol from daidzein [60]. Though all rats are equol producers, not all humans produce equol and equol producing capacity may change within an individual over time, possibly 2.5 years. [61]. Thus, race, menopausal stage, equol production, dose of soy isoflavone, and bone site may be factors to consider when assessing the effect of soy phytoestrogens in humans.

## 5. Soy Isoflavones, Vitamin D and Bone

**Figure 1:**
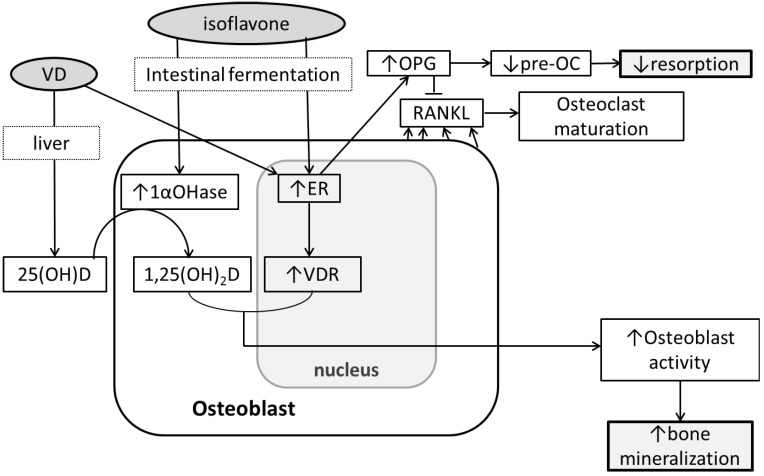
Possible mechanisms of the effect of soy isoflavones and vitamin D on bone metabolism. Soy isoflavones and vitamin D (VD) can synergistically stimulate bone formation through inducing 1,25-dihydroxyvitamin D synthesis and vitamin D receptor (VDR) expression. 1,25(OH)_2_D bound to VDR can induce osteoblast activity and bone formation. Also, bone resorption may be reduced through inducing estrogen receptor (ER) expression, which is affected by VD and soy isoflavones. An increase in ER can lead to an increase in OPG formation and thus prevents osteoclast (OC) maturation, resulting in less bone resorption.

The possibility of soy isoflavones and vitamin D impacting Ca and bone metabolism has been investigated through cell cultures and animal models. In intestinal cancer cells, genistein up-regulates VDR transcription and VDR expression possibly through the ER and MAPK signaling pathway [62] but no reported increase in Ca absorption [43]. One proposed mechanism is that phytoestrogens increase VDR and vitamin D metabolites in bone cells. It has been reported that estrogen upregulates VDR in osteoblast-like cells [63,64]. Also, genistein and daidzein increased the expression of CYP27B1 mRNA and suppressed CYP24 mRNA expression, the enzymes that activate and deactivate 1,25(OH)_2_D, respectively, in colon cancer cells [65]. Intake of cowpea isoflavones and 17β-estradiol independently affected vitamin D status, BMD and BMC in osteoporotic rats fed a low Ca low vitamin D diet (0.15% Ca and 0.1 IU vitamin D/day) [66]. In early postmenopausal women, 2-year soy isoflavone supplementation reduced the loss of spinal BMD only in those with 25(OH)D below 50 nmol/L [59]. The soy isoflavones may have stimulated 1,25(OH)_2_D production under conditions of the low levels of substrate 25(OH)D. Though *in vitro*, rodent, and clinical studies suggest a relationship, more evidence must be provided to test this hypothesis and elucidate the mechanism. Another proposed mechanism is that vitamin D analogs induce ER transcription. This has been reported in the JK 1624F_2_-2 (JFK) analog in pre- and post-menopausal women’s bone cells [65]. The synergistic effect of 1,25(OH)_2_D and 17β-estradiol increased osteoblastic MC3T3-E1 cell proliferation and viability [67]. Post-menopausal women receiving HRT and low amounts of vitamin D + Ca supplements (300 IU vitamin D + 93 mg Ca/day), but not those receiving HRT only, for 4 years had higher BMD compared to controls that did not receive HRT, vitamin D, nor Ca [68]. A similar effect by vitamin D and phytoestrogens may be possible, but no randomized controlled trial to investigate the synergistic effect of phytoestrogens and vitamin D has yet been reported. There may also be a threshold of the synergistic benefit of phytoestrogens and vitamin D as seen in OVX rats where genistein aglycones up to 54 mg/day human equivalent daily doses benefitted bone at standard Ca and vitamin D intakes, but not with higher Ca and vitamin D intakes [69]. Vitamin D and phytoestrogens may have a synergistic effect on bone (Figure 1) but the mechanism is still unclear and whether animal experiments can be translated into clinical effects has not been studied.

## 6. Conclusions

This review summarizes the evidence to support the possible benefit of the combination of vitamin D and soy phytoestrogens on post-menopausal bone loss. Clinical evidence for maximal vitamin D-mediated Ca absorption does not completely explain the optimal vitamin D status for maximal BMD or BMC and lacks correlation of vitamin D-related Ca absorption and BMD or BMC in post-menopausal women. The benefit of vitamin D on bone seems to be stronger through the direct effect on osteoblasts and osteoclasts. Soy phytoestrogens in combination with vitamin D may synergistically induce osteoblast activation and prevent pre-osteoclast and osteoclast differentiation, through the increase of vitamin D metabolites, VDR, and ER actions. More research is required to identify the causal factors and the mechanism of the benefits of vitamin D and phytoestrogens on bone in post-menopausal women.
